# Development of a wide field of view X-ray imaging detector and its application in X-ray multi-scale imaging

**DOI:** 10.1107/S1600577526009665

**Published:** 2026-10-12

**Authors:** Masato Hoshino, Kentaro Uesugi

**Affiliations:** a Japan Synchrotron Radiation Research Institute 1-1-1 Kouto Sayo Hyogo679-5198 Japan; Tohoku University, Japan

**Keywords:** X-ray multi-scale imaging, wide field of view imaging, X-ray imaging detector

## Abstract

A wide field of view X-ray imaging detector with an effective field of view of more than 90 mm has been developed. To enable distortion-free X-ray micro-imaging in the wide field of view observation, a high-definition telecentric lens was employed as visible-light optics after converting the incident X-ray beam into visible light. In the evaluation of the developed detector, it was confirmed that tomographic reconstruction could be performed without any noticeable distortion even at the edge of the reconstructed field of view. The developed wide field of view X-ray imaging detector was applied to X-ray multi-scale micro-tomography to capture the entire structure of relatively large objects such as fossils. By combining this with a local high-resolution observation, hierarchical structural visualization from the entire structure to the cellular level could be demonstrated.

## Abstract 2

A wide field of view X-ray imaging detector has been developed, and applied to wide-range X-ray multi-scale imaging to visualize a relatively large object from its entire structure to the cellular level.

## 1. Introduction

Synchrotron radiation based X-ray micro-tomography is an extremely powerful imaging technique for measuring the three-dimensional structures of samples with diameters of several millimetres to several tens of millimetres without destruction (Stampanoni *et al.*, 2007 ▸; Rack *et al.*, 2008 ▸; Uesugi *et al.*, 2010 ▸; Weitkamp *et al.*, 2022 ▸), and it is now used as a standard analytical tool in various research fields. Although progress is being made in increasing the brightness in next-generation synchrotron radiation facilities with lower emittance (Rack, 2020 ▸), some beamlines are still capable of measuring larger samples using a wide X-ray beam (Arhatari *et al.*, 2021 ▸; Gasilov *et al.*, 2024 ▸). The bending-magnet beamline BL20B2 at SPring-8 is one of the beamlines suitable for X-ray micro-imaging with a wide field of view (FOV) (Sera *et al.*, 2011 ▸; Imai *et al.*, 2019 ▸; Hoshino *et al.*, 2023 ▸). BL20B2 is a so-called medium-length beamline at SPring-8, and it has experimental hutches located at more than 200 m from the source, allowing the use of a collimated X-ray beam with a width of more than 100 mm (Goto *et al.*, 2001 ▸). This is a promising feature for capturing the entire structure of relatively large objects such as fossils in a single tomographic scan (Imai *et al.*, 2019 ▸). Even in such a large object, the local fine structure can be of great interest for further analysis. In this situation, a high-resolution observation of the local structure is desirable in addition to an observation of the entire structure, in order to understand the detailed structure. While the beam size determines the FOV, the flux density of the beam can be considered the most important parameter in determining the spatial resolution of the acquired image. From the perspective of the relationship between the flux density and the achievable spatial resolution, an undulator beam is much more adapted to high-resolution observation—for example, in nano-tomography based on X-ray microscopy—although its effective FOV is restricted to a very narrow region (De Andrade *et al.*, 2021 ▸; Takeuchi *et al.*, 2023 ▸). By comparison, the flux density in the wide X-ray beam used for wide-FOV observation tends to be lower than that of the undulator-based narrow and highly collimated X-ray beam. Therefore, improvement of the flux density in the wide X-ray beam is essential to achieve both high-resolution observation and wide-FOV observation. To compensate for the flux density in the wide X-ray beam from a bending-magnet source or a wiggler source, white beam or pink beam is generally used (Stampanoni *et al.*, 2006 ▸; Rack *et al.*, 2010 ▸; Koyama *et al.*, 2022 ▸; Arhatari *et al.*, 2025 ▸). Except for measurements that require a highly monochromated X-ray beam, X-ray micro-imaging using wide-band X-rays has become a common technique, especially in the high-energy X-ray region (Hoshino *et al.*, 2017 ▸). BL20B2 at SPring-8 employs double-multilayer monochromators (DMMs), each covering a different X-ray energy range (40 keV and 110 keV), to achieve both high flux density and large beam size (Koyama *et al.*, 2022 ▸), making it possible to support measurements ranging from high-resolution observation to wide-FOV observation at the same beamline. Therefore, X-ray multi-scale or hierarchical imaging can be a promising analytical technique for relatively large objects, taking advantage of the unique characteristics of this beamline. Several X-ray multi-scale imaging systems at synchrotron radiation facilities have already been reported (Mittone *et al.*, 2020 ▸; Velasco *et al.*, 2022 ▸; Bonnin *et al.*, 2024 ▸; Brunet *et al.*, 2024 ▸). For example, HiP-CT at ESRF is capable of visualizing human organs with cellular resolution (Walsh *et al.*, 2021 ▸). From the perspective of zooming in on the region of interest within a large object, X-ray multi-scale imaging should be composed of at least two scans: a wide-FOV observation to grasp the entire structural information quickly, and a local high-resolution observation of the region of interest identified from that overview. To achieve this type of ‘zooming-in’-based X-ray multi-scale imaging, an X-ray imaging detector capable of capturing the entire structure of a large object in a single scan is essential. In this study, the possibility of X-ray multi-scale imaging at BL20B2 at SPring-8 is presented, along with a description of the development of the wide-FOV X-ray imaging detector.

## 2. Development of the wide field of view X-ray imaging detector

**Figure 1:**
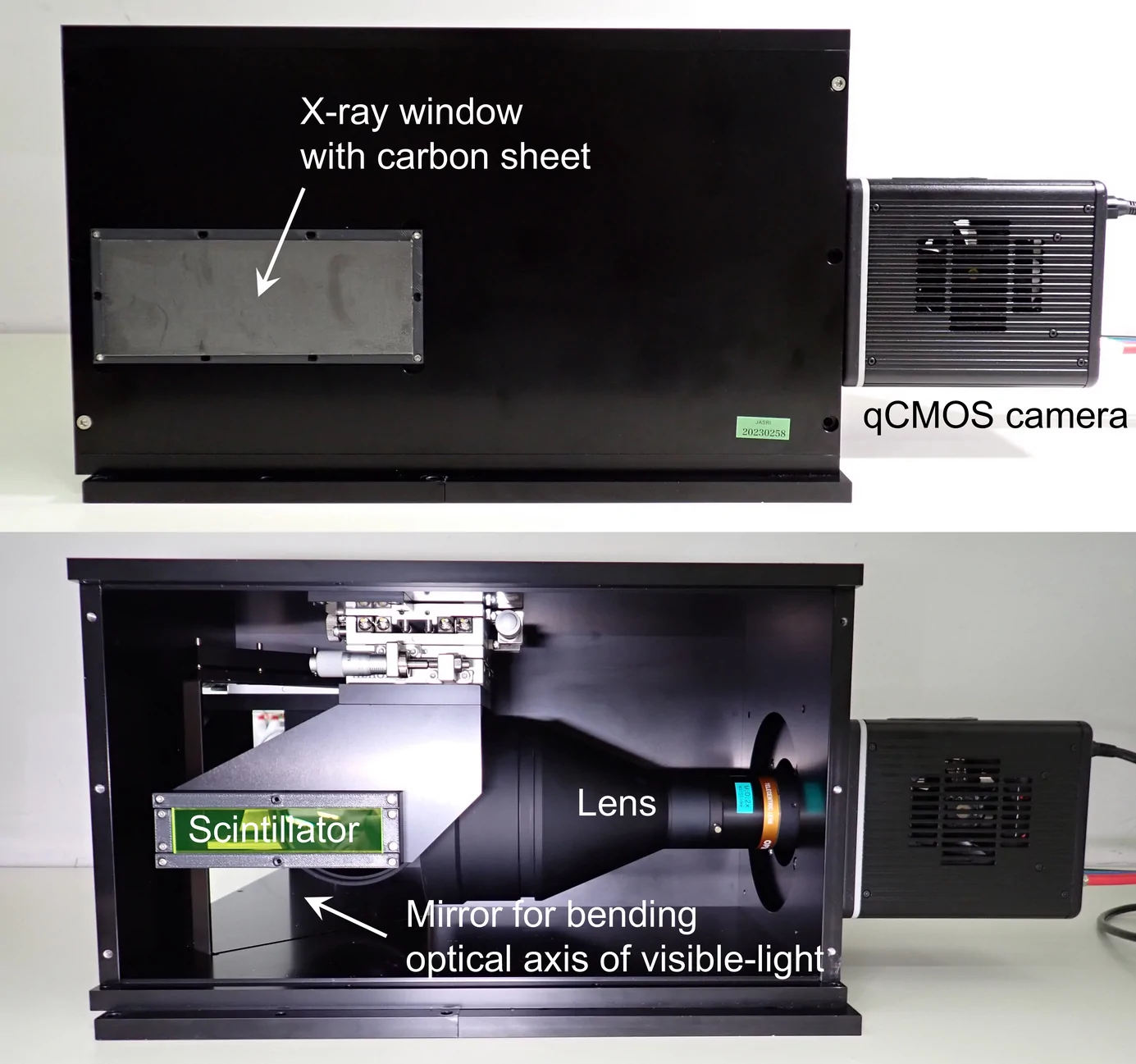
Photographs of a wide-FOV X-ray imaging detector employing a telecentric lens and a high-definition qCMOS camera; exterior view (top) and interior view (bottom).

In projection-based X-ray imaging, performance frequently depends strongly on the indirect X-ray imaging detector [see, for example, Bonse & Busch (1996 ▸) and Koch *et al.* (1998 ▸), for general background on indirect X-ray detection]. When detecting synchrotron radiation based high-intensity X-rays, a visible-light conversion-type X-ray imaging detector is generally used to avoid radiation damage to the camera sensor from strong X-ray irradiation. In this kind of detector, the X-ray image reaching the scintillator is converted into a visible-light image, which is then imaged onto the camera sensor via a lens system. In this case, the effective pixel size and the FOV can be adjusted over a wide range by choosing a suitable lens system, allowing measurements ranging from high-resolution observation to wide-FOV observation. Several types of lens systems exist: for instance, a tandem lens system has been used for relatively low-magnification imaging, and an objective lens system for high-magnification imaging (Uesugi *et al.*, 2012 ▸). We previously developed a wide-FOV detector for 200 keV high-energy X-ray micro-tomography using a tandem lens system comprising two camera lenses (Hoshino *et al.*, 2020 ▸); such a system is often subject to peripheral image distortion. In the present study, a one-sided high-definition telecentric lens for machine-vision use was instead employed, avoiding image distortion and yielding a clear, sharp image even in wide-FOV observations. The telecentric lens used (Model: FT02-150, OPTART, Japan) had an optical magnification of 0.2×, a numerical aperture of 0.0167 and optical distortion of less than 0.03%. This lens was attached to a high-definition qCMOS camera [ORCA Quest, Model: C15550-20UP, Hamamatsu Photonics, Japan; 4096 (H) × 2304 (V) pixels, native pixel size 4.6 µm] via a C-mount attachment. This setup was expected to give an effective pixel size of 23 µm and a FOV of 94 mm in width. An Lu_3_Al_5_O_12_(LuAG):Ce^+^ single-crystal scintillator (Crytur, Czech Republic), 100 mm × 25 mm in size and 500 µm thick, was used as the scintillating material. The exterior and interior views of the wide-FOV X-ray imaging detector developed are shown in Fig. 1 ▸. Since the optical axis of the visible light is bent by 90° in the horizontal plane by a plane mirror placed just behind the scintillator, the X-ray beam does not directly enter the camera sensor, allowing it to be used even in the high-energy X-ray region. The focus of the image was adjustable by moving the LuAG crystal along the optical axis of the X-ray beam.

**Figure 2:**
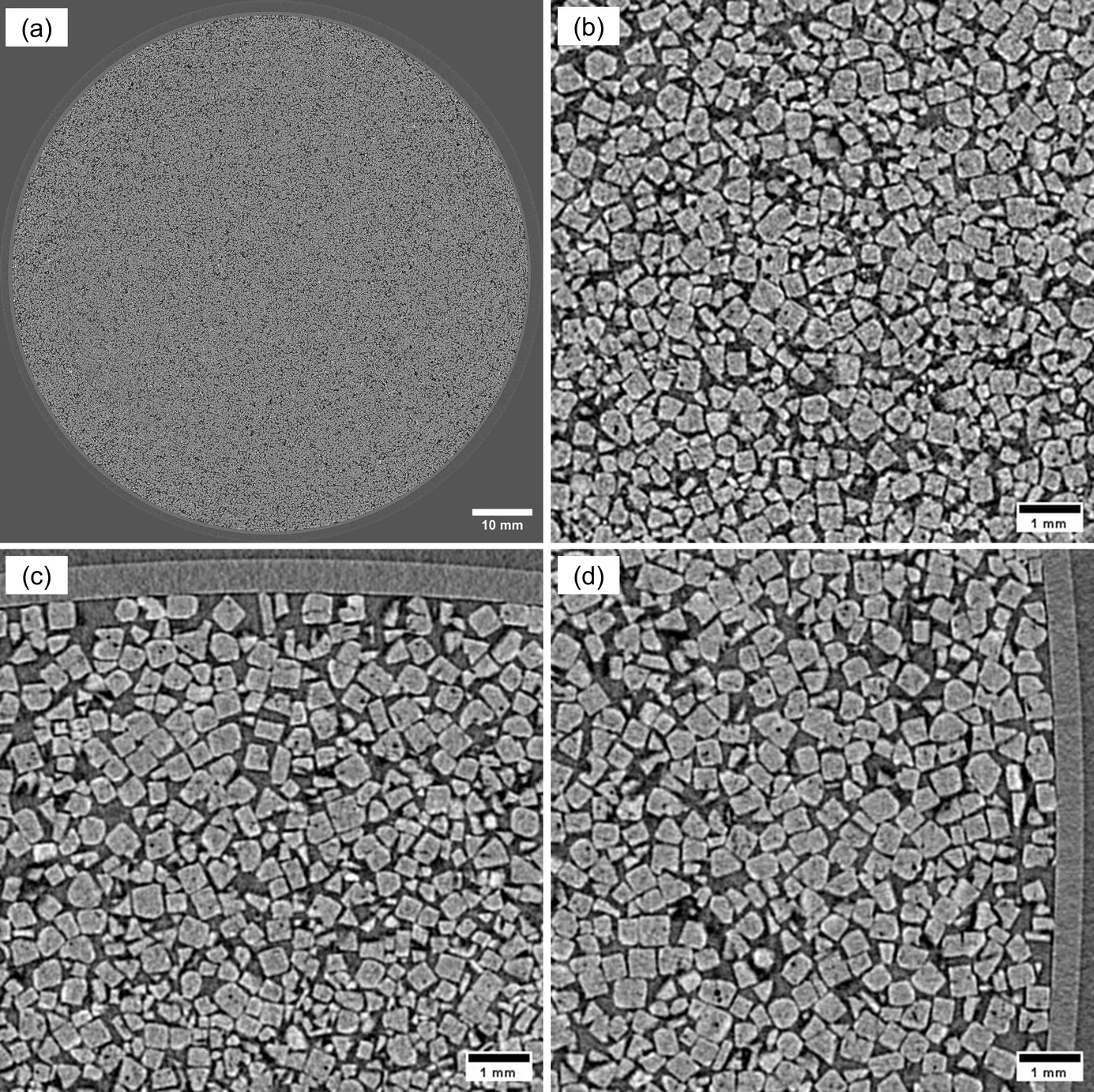
(*a*) Reconstructed sectional image (4096 × 4096 pixels) of sodium chloride particles filling a petri dish approximately 90 mm in diameter. Digitally enlarged images (400 × 400 pixels) at the (*b*) center, (*c*) top edge and (*d*) right edge parts in (*a*).

To evaluate the image distortion of the detector for tomographic use, a test sample containing small structures spanning almost the full width of the effective FOV was studied. In this case, the image quality of the sectional image was evaluated to determine whether the tomographic reconstruction could be performed without distortion, even at the edge of the effective FOV. This evaluation was conducted at experimental hutches 2 and 3 (EH2&3) of BL20B2, which is capable of using a wide beam combined with partially coherent X-rays. As a test sample, sodium chloride particles filling a petri dish approximately 90 mm in diameter were studied. The X-ray energy was set to 110 keV, available from the DMMs. The propagation distance from the sample to the detector was set to 8 m. The effective pixel size at the sample position was then measured to be 22.08 µm, slightly smaller than the expected value due to slight divergence of the X-ray beam during propagation. Thus, the effective FOV in the projection images was 90.4 mm in width, while the vertical FOV was set by the height of the incident beam at EH2&3 to approximately 10 mm—this is not a limitation of the detector’s active sensor area, which would support a substantially larger vertical FOV if a taller incident beam was available. A sectional image of the test sample, reconstructed by filtered back-projection using a Chesler filter (as for all tomographic reconstructions in this study), is shown in Fig. 2 ▸(*a*). The reconstructed image had a format of 4096 × 4096 pixels. Digitally enlarged images at the center, top edge and right edge, each a 400 × 400 pixel digital crop from Fig. 2 ▸(*a*), are shown in Figs. 2 ▸(*b*)–2(*d*), respectively. Significant distortion would be expected to manifest as artifacts such as streaks, with severity depending on the degree of distortion. As far as can be seen in the enlarged images, no significant difference could be observed at any position within the effective FOV. Therefore, it can be concluded that there is no significant image distortion within the effective FOV of 90.4 mm at an effective pixel size of 22.08 µm.

## 3. Application to X-ray multi-scale imaging

In X-ray micro-tomography, the effective FOV in the reconstructed sectional image is basically determined by the effective pixel size and the number of pixels of the camera sensor used. This means there is a trade-off between spatial resolution and effective FOV. One advantage of high-energy X-rays is that their high penetration power allows large objects to be imaged. Therefore, observing large objects can be a promising application of high-energy X-ray micro-imaging. When observing the entire structure, however, spatial resolution is limited by the trade-off described above, so that some important structures below the pixel size cannot be visualized. To overcome this and allow observation of both the entire structure and its fine structure, X-ray multi-scale imaging composed of a wide-FOV observation and a local high-resolution observation can be an effective technique.

**Figure 3:**
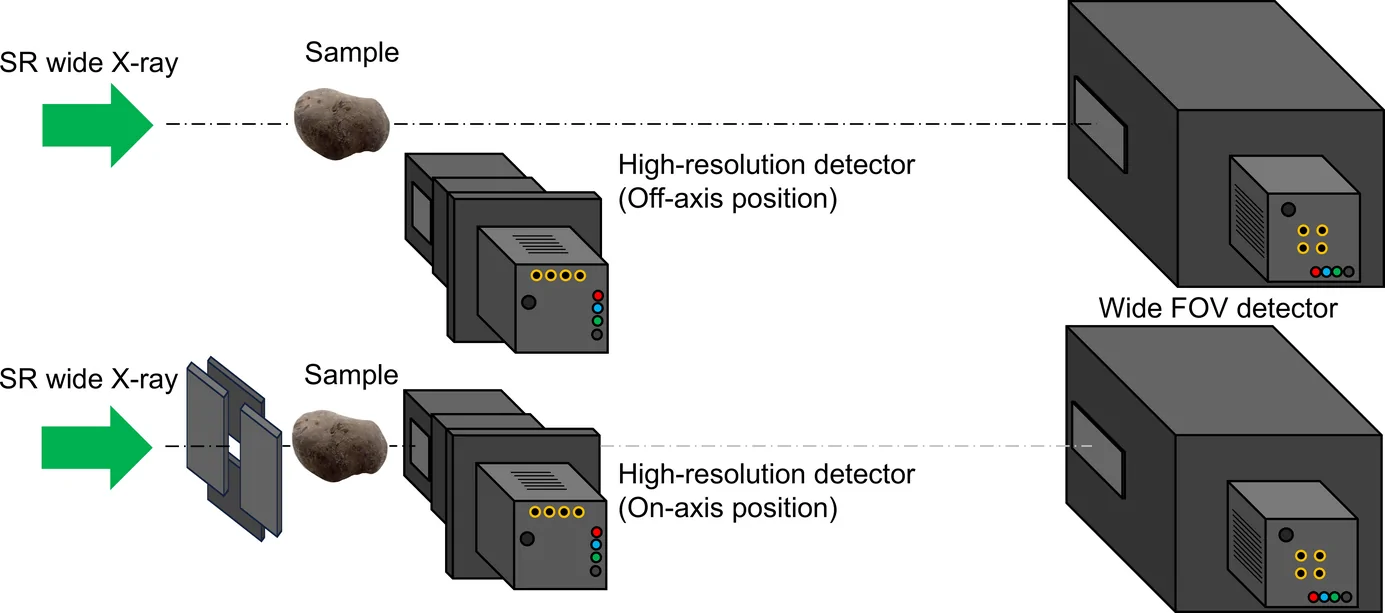
Schematic drawing of the basic concept of X-ray multi-scale imaging comprising a wide-FOV detector and a high-resolution detector; wide-FOV observation (top) and local high-resolution observation (bottom). In the high-resolution observation, a four-quadrant slit was set in front of the sample to avoid irradiation of the wide X-ray beam onto the high-resolution detector.

A basic concept of X-ray multi-scale imaging at BL20B2 is shown in Fig. 3 ▸. The wide-FOV observation and the local high-resolution observation can be switched by changing the X-ray imaging detector. The sample-to-detector distance was chosen, for each observation, from a simple practical estimate, *z* ≃ *p*^2^/λ (equivalently, a Fresnel number of order unity), where *p* is the effective pixel size and λ is the X-ray wavelength (Cloetens *et al.*, 1996 ▸; Momose, 2005 ▸). Here, the square root of *z*λ was kept larger than the effective pixel size for the local high-resolution observations to obtain sufficient edge enhancement for the inner structures, and smaller than the effective pixel size for the wide-FOV observations to observe the entire structure with moderate phase contrast. As mentioned above, the sample-to-detector distance needed for phase contrast increases with effective pixel size and X-ray energy, so the wide-FOV detector can generally be placed further from the sample, as shown in Fig. 3 ▸. By switching detectors without dismounting the sample from the rotation stage used for tomography, the local observation point can be easily identified from the coordinates of the wide-FOV image. Here, the wide-FOV X-ray imaging detector shown in Fig. 1 ▸ was used to demonstrate ‘zooming-in’-based X-ray multi-scale imaging for relatively large objects.

**Figure 4:**
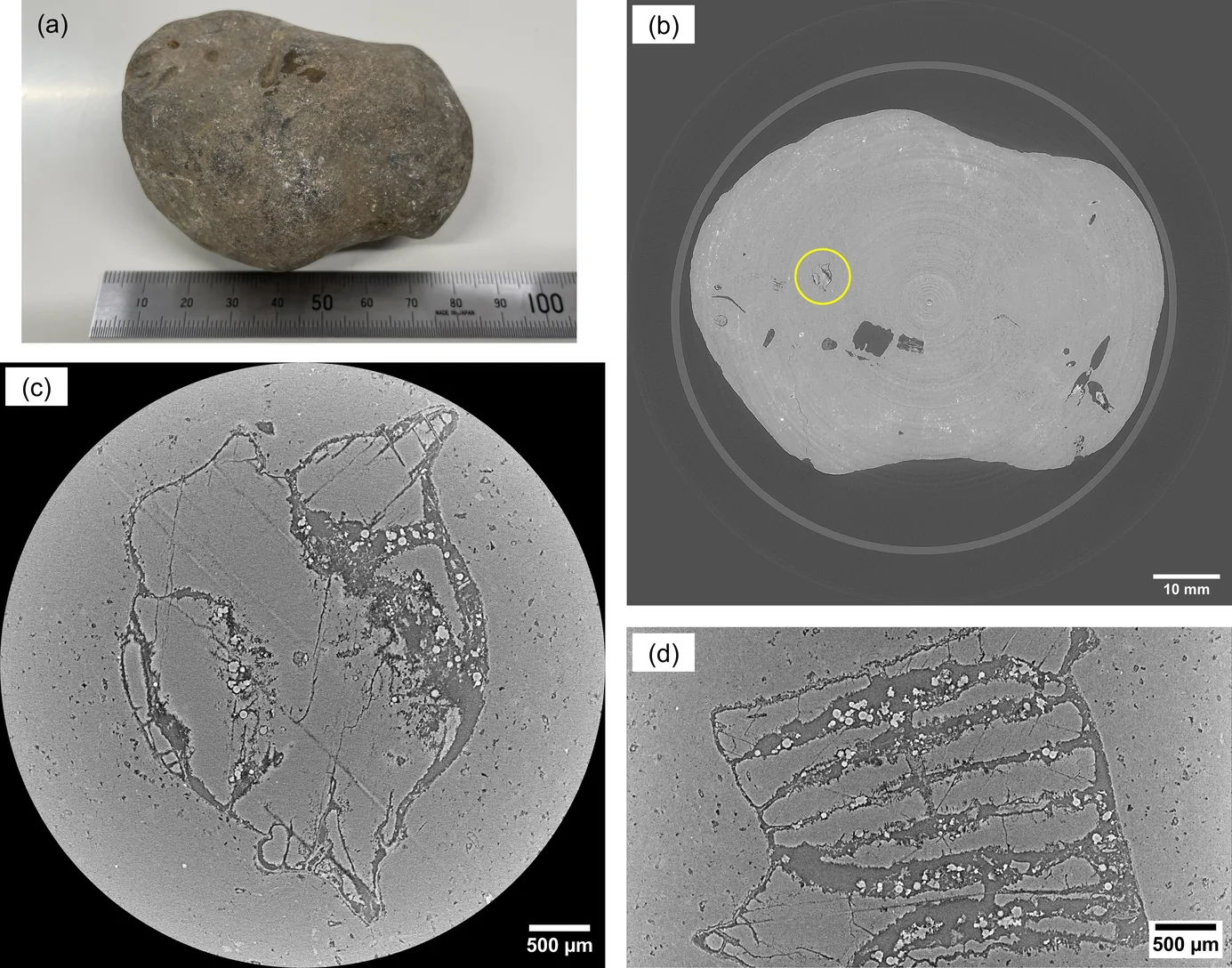
X-ray multi-scale micro-tomography of a nodule. (*a*) Photograph of the nodule. (*b*) Wide-FOV sectional image of the entire structure of the nodule. (*c*) Local high-resolution sectional image at the circle shown in (*b*). (*d*) Sectional image perpendicular to the image shown in (*c*).

As a demonstration of X-ray multi-scale micro-tomography, a fossil-like sample, a nodule, was studied. A photograph of the nodule is shown in Fig. 4 ▸(*a*). First, the entire structure was observed with the wide-FOV detector, under the following measurement conditions: X-ray energy 110 keV, propagation distance 8 m, effective pixel size 22.08 µm, number of projections 3600, exposure time 120 ms per image. A sectional image of the nodule is shown in Fig. 4 ▸(*b*). Some voids and inclusions can be identified. Among these, a small inclusion, highlighted by the yellow circle, was studied by high-resolution detector. The high-resolution X-ray imaging detector used consisted of a visible-light-conversion-type X-ray imaging detector employing a tandem camera lens system: an AI AF Nikkor 35 mm *f*/2*D* lens (Nikon, Japan; focal length 35 mm) and an AI AF Nikkor 180 mm *f*/2.8*D* IF-ED lens (Nikon, Japan; focal length 180 mm) were used as the first and second camera lenses, respectively, giving an optical magnification of 5.14×. A transparent LuAG ceramic scintillator, 200 µm thick, was used as the scintillating material. This is a distinct material from the LuAG single crystal used elsewhere in this study, produced by transparent-ceramics technology (see also Kameshima *et al.*, 2019 ▸). The magnified visible-light image was detected by a scientific CMOS camera (ORCA Lightning, C14120-20P, Hamamatsu Photonics, Japan; 4608 (H) × 2592 (V) pixels, native pixel size 5.5 µm). The measurement conditions for the local high-resolution observation were: propagation distance 1.5 m, effective pixel size 1.08 µm, number of projections 3600, exposure time 1 s per image. The high-resolution sectional image is shown in Fig. 4 ▸(*c*); Fig. 4 ▸(*d*) shows a sectional image perpendicular to that in Fig. 4 ▸(*c*). To reduce noise in these images, ten consecutive slice images were averaged. Since local (region-of-interest) tomography is subject to a cupping artifact arising from projection truncation at the boundary of the scanned FOV, the sectional image in Fig. 4 ▸(*c*) was reconstructed after applying a correction for the truncated projections (Ohnesorge *et al.*, 2000 ▸), which reduced this artifact. The detailed structure that could not be observed in the entire-structure image was clearly visualized.

**Figure 5:**
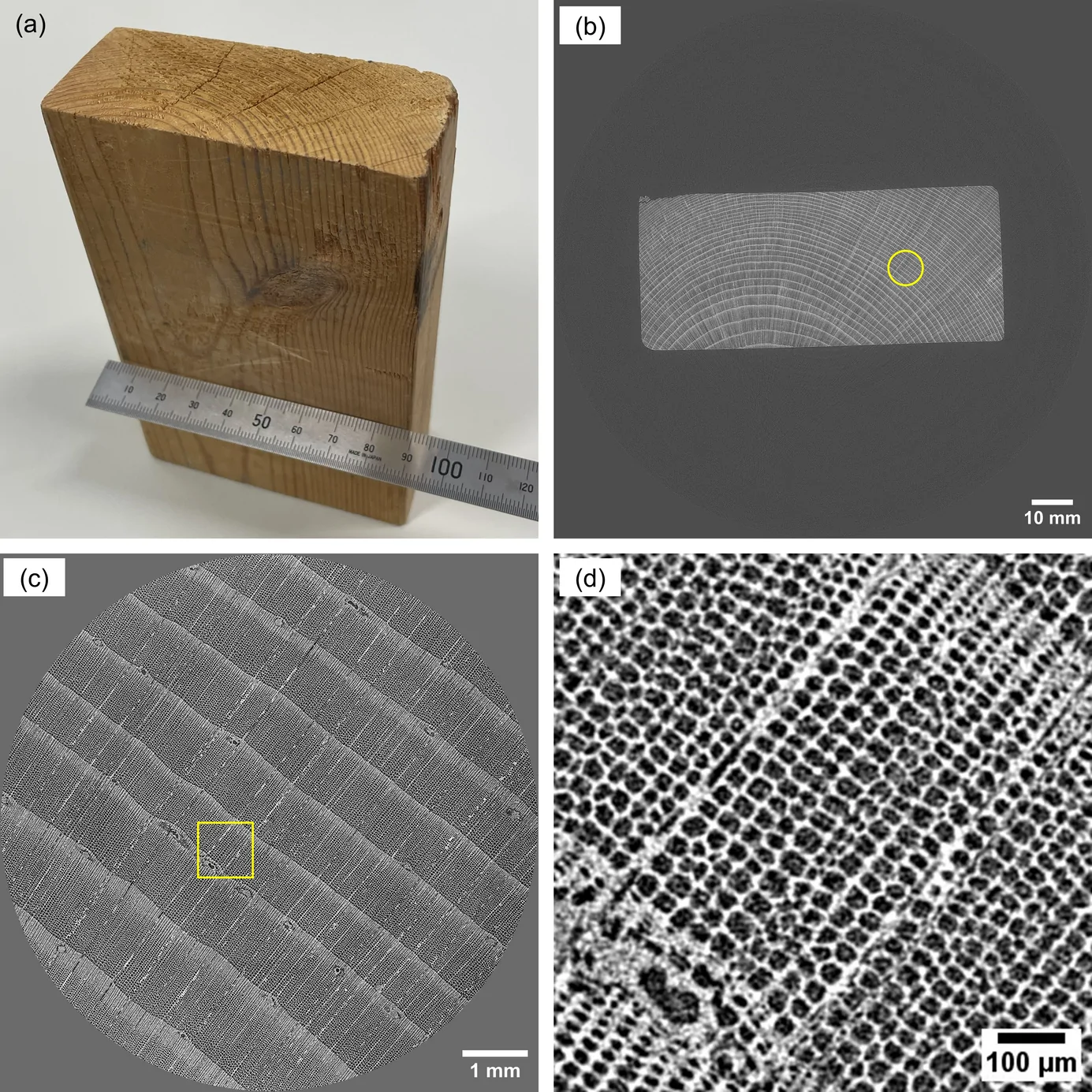
X-ray multi-scale micro-tomography of a piece of lumber. (*a*) Photograph of the lumber. (*b*) Wide-FOV sectional image of the entire structure of the lumber observed with an offset scan. (*c*) Local high-resolution sectional image at the circle shown in (*b*). (*d*) Digitally enlarged image of the square region shown in (*c*).

As a further demonstration of X-ray multi-scale micro-tomography, an offset scan was employed in the wide-FOV observation to enable observation of larger objects. In an offset scan using an almost parallel beam, the effective FOV in the reconstructed sectional image can be expanded to a maximum of just under twice the original value by shifting the rotation axis from the center to the edge of the projection image. This means that the effective FOV in the wide-FOV observation could be expanded to as much as 180 mm in this case. Here, a piece of lumber, shown in Fig. 5 ▸(*a*), was studied to visualize a multi-scale wooden structure. Because wood contains a mixture of structures ranging from annual rings down to individual cells, it is well suited to demonstrating the performance of the imaging system. To observe the entire structure of the lumber, an offset scan with a 20 mm offset of the rotation axis was used, giving an expected FOV of 130 mm, under the following measurement conditions: X-ray energy 110 keV, propagation distance 8 m, effective pixel size 22.08 µm, number of projections 3600, exposure time 150 ms per image. To acquire the projection images needed for the offset rotation axis, the sample was rotated 360°, and the two halves of each projection were stitched at the rotation axis to form expanded projection images. The reconstructed sectional image of the lumber is shown in Fig. 5 ▸(*b*). Although the annual rings are clearly visualized, the cellular structures cannot be observed. To see the smallest structures of the lumber, the region surrounded by the circle was studied with the high-resolution detector; in this case, another high-resolution detector employing a tandem camera lens system was used. A beam monitor, AA60 (Hamamatsu Photonics, Japan), including an X-ray scintillator and a first camera lens with focal length 75 mm, was used together with a second camera lens, Milvus 2/135 ZF.2 (Zeiss, Germany; focal length 135 mm), giving an expected optical magnification of 1.8×. A LuAG single crystal, 500 µm thick, was used as the scintillating material. The magnified visible-light image was detected with a high-definition CMOS camera [C13949-50U, Hamamatsu Photonics, Japan; 4096 (H) × 3008 (V) pixels, native pixel size 3.45 µm]. The measurement conditions for the high-resolution observation were: propagation distance 500 mm, effective pixel size 2.01 µm, number of projections 3600, exposure time 1 s per image. The local high-resolution sectional image is shown in Fig. 5 ▸(*c*); a digitally enlarged image of the square region is shown in Fig. 5 ▸(*d*) for detail. A low-pass filter (3D Gaussian Blur plugin in *ImageJ*; σ_*x*_ = σ_*y*_ = σ_*z*_ = 1.0 where σ is the standard deviation of the Gaussian) was applied to the local image in order to suppress random noise. The cellular structure characterizing each type of wood could be clearly visualized.

## 4. Wide-range X-ray multi-scale imaging

**Figure 6:**
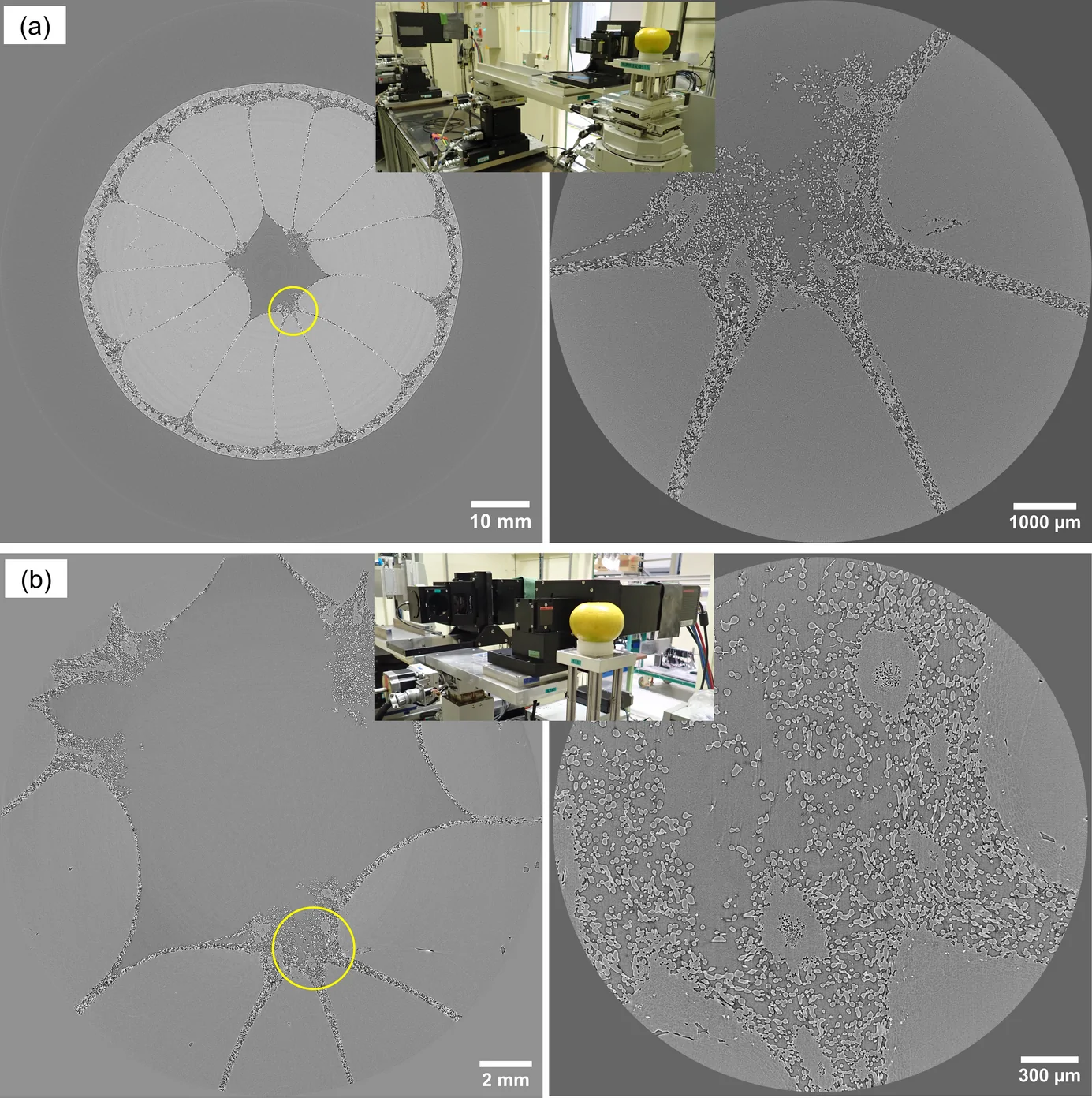
Wide-range X-ray multi-scale micro-tomography of a mandarin orange. (*a*) Wide-FOV sectional image (left) and local high-resolution sectional image at the circle (right) observed at EH2&3. (*b*) Wide-FOV sectional image (left) and local high-resolution sectional image at the circle (right) observed at EH1. Insets show photographs of the actual setups for the multi-scale observations.

**Figure 7:**
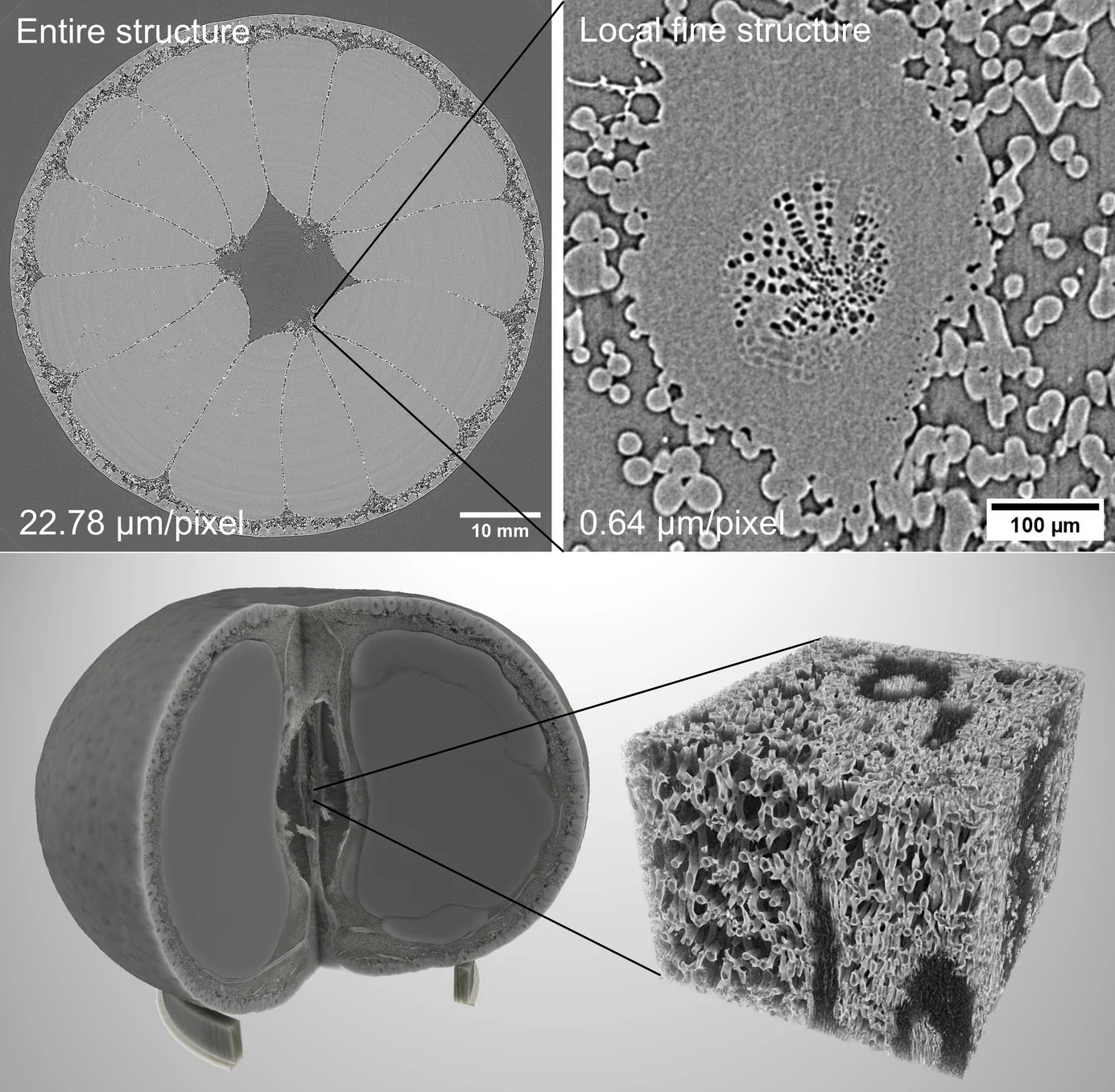
Upper images show the entire structure (left) and the local fine structure observed at the highest spatial resolution (right), obtained from wide-range X-ray multi-scale micro-tomography. Lower images show three-dimensional views based on the tomographic dataset shown above.

**Figure 8:**
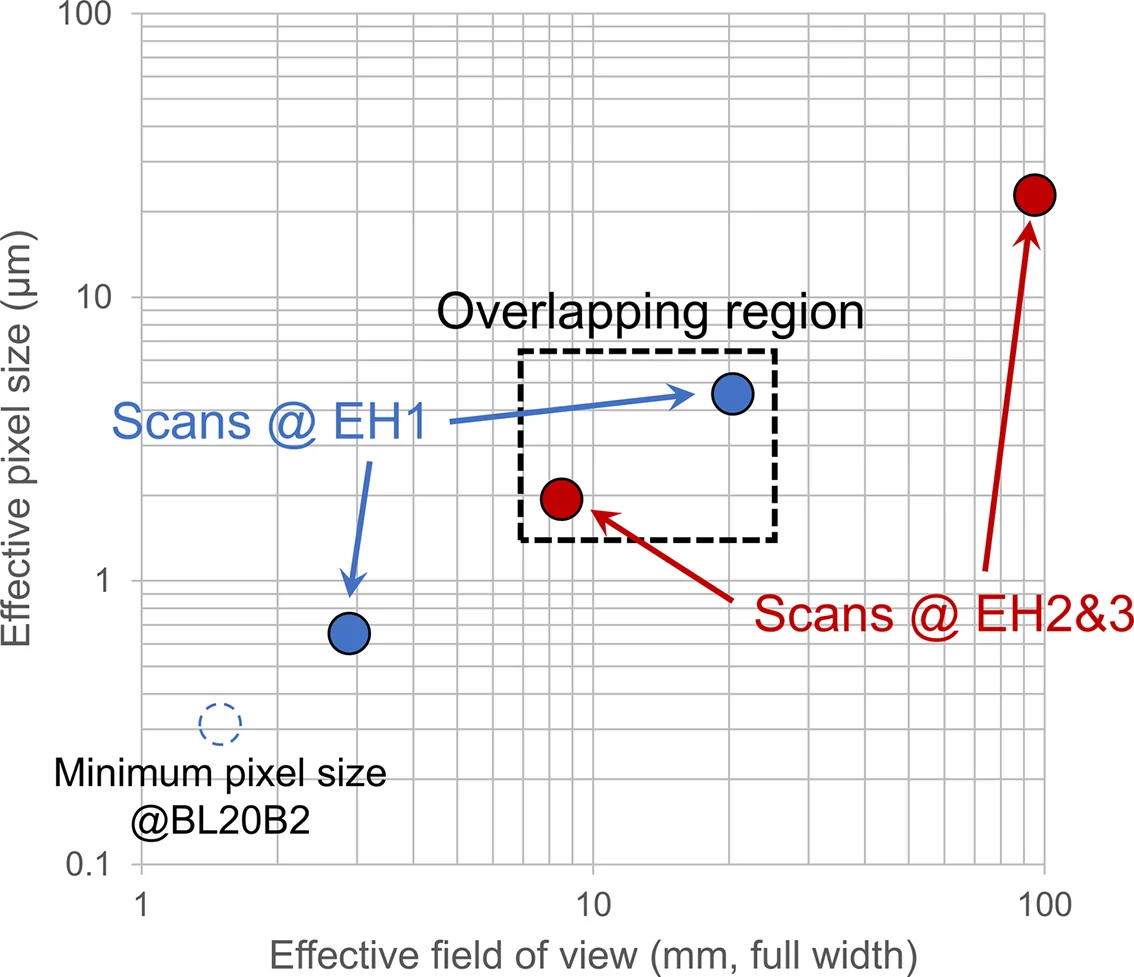
Relationship between the effective pixel size and the effective FOV in X-ray multi-scale imaging at BL20B2. Solid circles show the measurement conditions for the sectional images shown in Fig. 6 ▸. The minimum pixel size available at BL20B2 is shown as a dashed circle.

For the X-ray multi-scale imaging shown in Figs. 4 ▸ and 5 ▸, the large X-ray beam available at the endstation of BL20B2 was used for the wide-FOV observations. In the case of a bending-magnet source, however, a higher X-ray photon flux density is available at the experimental hutch closer to the source, while the effective beam size becomes smaller. This higher flux density makes it feasible to use higher-magnification optics in the indirect X-ray imaging detector, *i.e.* a smaller effective pixel size. BL20B2 has an upstream experimental hutch (EH1) located at 43 m from the source, inside the SPring-8 storage ring building, unlike EH2&3, which are located in a separate building away from the storage ring building. Therefore, it is possible to use an X-ray beam with approximately 25 times higher photon flux density at EH1 than at EH2&3, which in turn enables high-resolution X-ray micro-imaging with a sub-micrometre pixel size. Here, wide-range X-ray multi-scale imaging—a hybrid measurement combining a high-resolution observation at EH1 with a wide-FOV observation at EH2&3—was demonstrated. Although the sample must be dismounted from the sample stage for measurement at different experimental stations of the beamline, it is technically possible to observe the same sample without significant positional misalignment by using an improved sample-mounting method. In addition, by matching the effective pixel size and FOV so that the measurement ranges at EH1 and EH2&3 overlap, the datasets acquired at the two experimental stations can be directly compared and combined. As a demonstration of wide-range X-ray multi-scale micro-tomography, a mandarin orange was observed from its entire structure down to its cellular structure, through four-stage multi-scale observations at different experimental stations of the beamline. The measurement conditions for each observation are summarized in Table 1 ▸. The X-ray energy was set to 40 keV. The multi-scale sectional images of the mandarin orange measured at EH2&3 and EH1 are shown in Figs. 6 ▸(*a*) and 6 ▸(*b*), respectively. A low-pass filter (3D Gaussian Blur plugin in *ImageJ*; σ_*x*_ = σ_*y*_ = σ_*z*_ = 1.0) was applied to the local sectional images. As shown in Fig. 6 ▸(*b*), a reconstructed FOV of more than 20 mm could be obtained even at EH1, meaning that an overlapping region from 10 mm to 20 mm in effective FOV was achieved between the observations at EH1 and EH2&3. A magnified image of the square region in the highest-resolution image, measured with an effective pixel size of 0.64 µm, is shown in Fig. 7 ▸, together with the entire sectional image for reference. Even the vascular bundles could be clearly visualized. The scale bars in Fig. 7 ▸ differ by approximately a factor of 100. Three-dimensional views from the entire structure to its local fine structure are also shown at the bottom of Fig. 7 ▸, rendered using *Drishti* (Limaye, 2012 ▸). The microscopic networks in the central part of the mandarin orange are clearly visualized. The images in Figs. 6 ▸ and 7 ▸ are shown without phase retrieval, so as to present the images exactly as measured. Fig. 8 ▸ summarizes the relationship between the effective pixel size and the FOV that can be covered at the different experimental stations of the beamline, based on the knowledge gained from wide-range X-ray multi-scale micro-tomography. The measurement conditions for the sectional images shown in Fig. 6 ▸ are plotted as solid circles, and the overlapping region in effective pixel size and effective FOV between the observations at EH1 and EH2&3 is indicated by a dotted rectangle. This result implies that X-ray multi-scale imaging conducted at different experimental stations of the beamline has the potential to provide seamless observation over two orders of magnitude of spatial scale.

## 5. Further evaluation of detector performance

**Figure 9:**
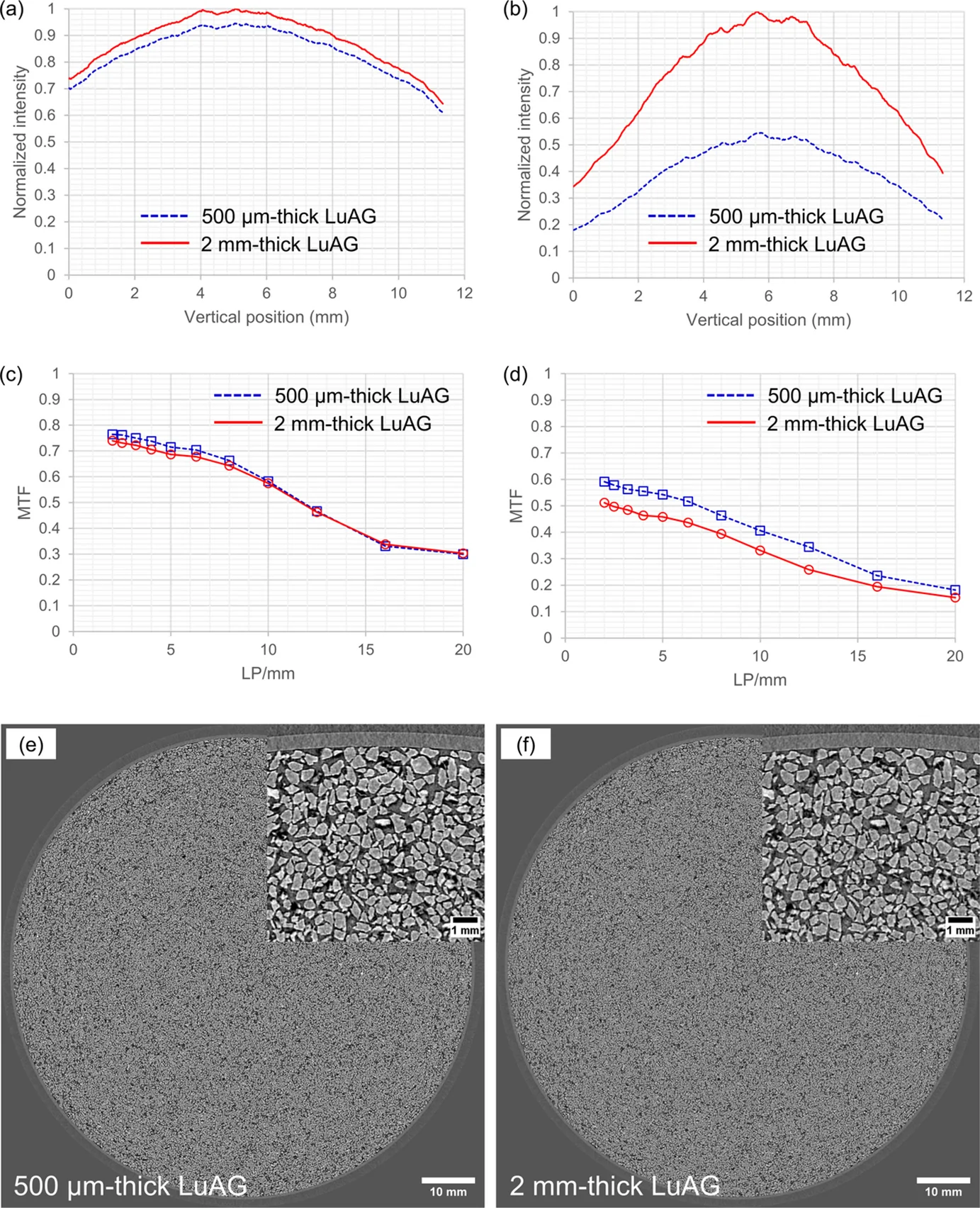
Vertical intensity profiles of (*a*) 40 keV and (*b*) 110 keV X-ray beams, measured with the developed wide-FOV X-ray imaging detector. Modulation transfer functions of the wide-FOV X-ray imaging detector, measured with (*c*) 40 keV and (*d*) 110 keV X-ray beams. Reconstructed sectional images of silica sand filling a petri dish, observed with (*e*) 500 µm-thick and (*f*) 2 mm-thick LuAG crystals.

The wide-FOV X-ray imaging detector developed in this study could acquire a tomographic image with an effective FOV of more than 90 mm in a single scan, using a distortion-free telecentric lens combined with a high-definition CMOS camera. Further expansion of the effective FOV would in principle be possible by employing a larger-diameter lens, since the maximum beam width at the endstation of BL20B2 is approximately 300 mm (Koyama *et al.*, 2022 ▸). A notable advantage of the single-scan approach is its potential for application to dynamic observation. By optimizing the measurement conditions, a shorter scan and potentially four-dimensional observation should be achievable. In this case, detector efficiency is an important factor in achieving a shorter scan. In this study, a LuAG single crystal, 500 µm thick, was used as the X-ray scintillator in the wide-FOV detector developed. To improve efficiency, the use of a thicker crystal can be considered, taking into account the depth of field of the telecentric lens used. In particular, for the high-energy X-ray micro-imaging above 100 keV described in this study, an increase in visible-light emission from a thicker crystal would be expected, since high-energy X-rays have a large penetration depth even in dense scintillating materials. Ideally, an improvement in efficiency while maintaining spatial resolution and image contrast would be expected by properly exploiting the increased emission from a thicker crystal. To verify this possibility, the image intensity (*i.e.* the conversion efficiency from X-rays to visible light), the modulation transfer function (MTF) and the tomographic image quality were evaluated for LuAG single crystals 500 µm and 2 mm thick. First, to evaluate the efficiency, the intensity along the vertical direction of the beam was measured. Vertical beam intensity profiles for X-ray energies of 40 keV and 110 keV are shown in Figs. 9 ▸(*a*) and 9 ▸(*b*), respectively. Although no marked difference was observed at 40 keV, approximately twice the efficiency was observed at 110 keV. This behavior can be explained by the transmittance of the LuAG crystals: at 40 keV, transmittance is 13.9% for the 500 µm crystal and 0.04% for the 2 mm crystal; at 110 keV, transmittance is 51.4% for the 500 µm crystal and 7.0% for the 2 mm crystal. Most 40 keV X-rays are absorbed even by the 500 µm crystal, whereas, at 110 keV, almost half the X-rays penetrate the 500 µm crystal, and most are absorbed by the 2 mm crystal. Next, the MTFs were measured using a test chart (Moriyama X-ray Equipments Co. Ltd, Japan). Measured MTFs for X-ray energies of 40 keV and 110 keV are shown in Figs. 9 ▸(*c*) and 9 ▸(*d*), respectively. As with the efficiency, no difference was observed at 40 keV. At 110 keV, contrast was somewhat degraded for the thicker crystal, possibly due to an increase in background not contributing to image formation. Finally, tomographic images were acquired using both LuAG crystals, under the following measurement conditions: X-ray energy 110 keV, propagation distance 8 m, effective pixel size 22.12 µm, number of projections 3600. Exposure time was set so that the background intensity was approximately the same for each crystal (150 ms per image for the 500 µm crystal, 80 ms per image for the 2 mm crystal). Silica sand with a particle diameter of around 0.6 mm was used as the test sample, filling a petri dish approximately 90 mm in diameter, as in Fig. 2 ▸. The tomographic sectional images obtained with the 500 µm and 2 mm crystals are shown in Figs. 9 ▸(*e*) and 9 ▸(*f*), respectively, with insets showing digitally enlarged images at the top of each sectional image. No significant difference could be observed. This qualitative visual comparison of efficiency, MTF and tomographic image quality indicates that the thicker scintillator crystal can be used without practical difficulty. It offers the potential to shorten scan time, at the cost of a slight decrease in projection-image contrast.

## 6. Outlook

From the results of X-ray multi-scale micro-tomography for several samples, this imaging technique was confirmed to have great potential for observing relatively large objects, from their entire structure down to microscopic structures. Although only X-ray micro-tomography was demonstrated in this study, X-ray multi-scale micro-lamino­graphy would also be a promising analytical tool for large planar objects such as fossils, metallic objects and electrical devices. In our previous study, preliminary multi-scale observations based on X-ray micro-lamino­graphy for planar fossils have already been demonstrated (Hoshino *et al.*, 2023 ▸). Using the wide-FOV X-ray imaging detector established in this study, we expect applications in visualizing the detailed structure of much larger planar fossils through multi-scale observation.

The propagation-based phase contrast (edge enhancement) used throughout this study depends on the sample-to-detector distance, and, while useful for visualizing weakly absorbing structures, stronger edge enhancement can also introduce artifacts near sharp sample boundaries. Incorporating a mechanism to freely vary the sample-to-detector distance at each experimental hutch would allow future measurements to be tuned, as needed for a given sample, between strong edge enhancement and near-absorption-only contrast, and is considered a worthwhile improvement for the imaging system.

## 7. Conclusion

A wide-FOV X-ray imaging detector employing a telecentric lens was developed, and it was confirmed that an effective FOV of more than 90 mm could be achieved without distortion in the tomographic sectional image. The wide-FOV detector developed was incorporated into an X-ray multi-scale imaging scheme, and X-ray multi-scale micro-tomography visualizing a relatively large object from its entire structure to microscopic structure was successfully demonstrated. Furthermore, the possibility of observing an object across two orders of magnitude of spatial scale was demonstrated using wide-range X-ray multi-scale imaging at different experimental stations of the beamline. In future, we expect to apply this technique to actual samples such as large fossils, biological samples and metallic objects, visualizing them from their entire structure down to cellular and metallographic detail.

## Figures and Tables

**Table 1:** Measurement conditions in wide-range X-ray multi-scale micro-tomography at different experimental stations of the beamline

	Magnification factor of lens	Propagation distance (mm)	Effective pixel size (µm)	Effective FOV (mm)	Number of projections	Exposure time (ms)
EH2&3 wide field of view	0.2×	2000	22.78	93.3	7200	6
EH2&3 local	2.4×	200	1.95	8.6	3600	40
EH1 wide field of view	1.0×	465	4.68	20.7	7200	1.5
EH1 local	7.0×	100	0.64	2.8	3600	200
